# Biomedical word sense disambiguation with ontologies and metadata: automation meets accuracy

**DOI:** 10.1186/1471-2105-10-28

**Published:** 2009-01-21

**Authors:** Dimitra Alexopoulou, Bill Andreopoulos, Heiko Dietze, Andreas Doms, Fabien Gandon, Jörg Hakenberg, Khaled Khelif, Michael Schroeder, Thomas Wächter

**Affiliations:** 1 Biotechnology Center (BIOTEC), Technische Universität Dresden, 01062, Dresden, Germany; 2 INRIA Sophia Antipolis, 2004 Route des Lucioles, 06902, Sophia Antipolis, France

## Abstract

**Background:**

Ontology term labels can be ambiguous and have multiple senses. While this is no problem for human annotators, it is a challenge to automated methods, which identify ontology terms in text. Classical approaches to word sense disambiguation use co-occurring words or terms. However, most treat ontologies as simple terminologies, without making use of the ontology structure or the semantic similarity between terms. Another useful source of information for disambiguation are metadata. Here, we systematically compare three approaches to word sense disambiguation, which use ontologies and metadata, respectively.

**Results:**

The 'Closest Sense' method assumes that the ontology defines multiple senses of the term. It computes the shortest path of co-occurring terms in the document to one of these senses. The 'Term Cooc' method defines a log-odds ratio for co-occurring terms including co-occurrences inferred from the ontology structure. The 'MetaData' approach trains a classifier on metadata. It does not require any ontology, but requires training data, which the other methods do not. To evaluate these approaches we defined a manually curated training corpus of 2600 documents for seven ambiguous terms from the Gene Ontology and MeSH. All approaches over all conditions achieve 80% success rate on average. The 'MetaData' approach performed best with 96%, when trained on high-quality data. Its performance deteriorates as quality of the training data decreases. The 'Term Cooc' approach performs better on Gene Ontology (92% success) than on MeSH (73% success) as MeSH is not a strict is-a/part-of, but rather a loose is-related-to hierarchy. The 'Closest Sense' approach achieves on average 80% success rate.

**Conclusion:**

Metadata is valuable for disambiguation, but requires high quality training data. Closest Sense requires no training, but a large, consistently modelled ontology, which are two opposing conditions. Term Cooc achieves greater 90% success given a consistently modelled ontology. Overall, the results show that well structured ontologies can play a very important role to improve disambiguation.

**Availability:**

The three benchmark datasets created for the purpose of disambiguation are available in Additional file 1.

## Background

Word sense disambiguation (WSD) deals with relating the occurrence of a word in a text to a specific meaning, which is distinguishable from other meanings that can potentially be related to that same word [1]. WSD is essentially a classification problem: given an input text and a set of sense tags for the ambiguous words in the text, assign the correct senses to these words. Sense assignment often involves two assumptions: *a*. within a discourse, e.g. a document, a word is only used in one sense [2] and *b*. words have a tendency to exhibit only one sense in a given collocation – neighbouring words [3].

During the last years, word sense disambiguation has become a hot topic in the biomedical domain. The challenge here is the rapid growth of the biomedical literature in terms of new words and their senses, with the situation getting worse with the use of abbreviations and synonyms. This illustrates the exact need in the case of the biomedical domain; the development of statistical approaches that utilize "established knowledge" (like thesauri, dictionaries, ontologies and lexical knowledge bases) and require no or only some parsing of the text in order to perform the correct annotation.

Two main decision points for WSD in the biomedical domain are the granularity to which WSD should be performed and the selection of an appropriate corpus for training and evaluation. Concerning granularity, some tasks are easier than others (e.g. distinguishing between 'bank' as a building vs the 'BANK' gene is easier than 'BANK' gene vs the protein). Concerning the biomedical corpora, those are few, mainly due to the time-consuming and labor-intensive process of manual or semi-automatic annotation. Examples of such datasets are the NLM WSD test collection [4], Medstract for acronyms [5] and the BioCreAtIvE set for mouse, fruitfly, and yeast [6]. However, depending on the task, researchers need to collect their own gold standard datasets.

### Algorithms for Word Sense Disambiguation

As shown in Table 1, WSD algorithms can be distinguished as *supervised*, *unsupervised*, or using *established knowledge *[1,7]. In the biomedical domain researchers have focused on supervised methods [8-11] and using established knowledge [12-15] to perform gene name normalization and resolve abbreviations. According to the recent BioCreAtIvE challenge, the former problem can be solved with up to 81% success rate [14] for human genes, which are challenging with 5.5 synonyms per name (therefore many genes are named identically).

**Table 1:** Algorithms for Word Sense Disambiguation.

	**publ**.	**Data**	**Background knowledge**	**Approach**	**Experiment**	**Accuracy**
*Established Knowledge*	[12]	gene definition & abstract vector	5 human gen. dbs & MeSH	cosine similarity	52,529 Medline abstracts, 690 human gene symbols	92.7%
	[13]	free text	UMLS, Journal Descriptors	Journal Descriptor Indexing (JDI)	45 ambiguous UMLS terms (NLM WSD Collection)	78.7%
	[14]	Medline abstracts	BioCreative-2 GN lexicon & text, EntrezGene, UniProt, GOA	motifs from multiple sequence alignments	BioCreative-2 GN challenge	81%
	[15]	Medline abstracts	list of gene senses, EntrezGene	inverse co-author graph	BioCreative GN challenge	97%P

*Supervised*	[8]	XML tagged abstracts, positional info, PoS	-	naive Bayes, decision trees, inductive rule training	protein/gene/mRNA assignment: 9 million words (mol. biol. journals)	85%
	[49]	text	-	word count, word cooc	-	86.5%
	[9,50]	Medline abstracts	UMLS terms	UMLS term cooc	35 biomedical abbreviations	93%P
	[10]	abbreviations in Medline abstracts	-	SVM	build dictionary, use for abbreviations occurring with their long forms	98.5%
	[11]	gene symbol context (*n *words +/-)	-	SVM	-	85%

*Unsupervised*	[19,20]	document	-	LSA/LSI, 2^*nd *^order cooc	170,000 documents, 1013 terms (TREC-1) (Wall Street Journal)	↑ 7–14%
	[51]	word cooc, PoS tags	WordNet	average link clustering	13 words, ACL/DCI	73.4%
	[21]				Wall Street Journal Corpus	
	[22]	-	-	1^*st*^, 2^*nd *^order context vectors (coocs within 5 positions)	24 Senseval-2 words, *Line*, *Hard*, *Serve *corpora	44%
	[23]	text	few tagged data, WordNet	co-training, collocations	12 common Engl. words × 4000 instances	96.5%
	[25]	-	-	co-training & majority voting	Senseval-2 generic English	↑ 9.8%
	[24]	-	WordNet	noun coocs, Markov clustering	-	-

Resolving ambiguous abbreviations achieves higher success rates of close to 100%, as the task is less complex when long forms of the abbreviated terms are in the document [10]. The above approaches use cosine similarity [12], SVM [10,11], Bayes, decision trees, induced rules [8], and background knowledge sources such as the Unified Medical Language System (UMLS) [16], Medical Subject Headings (MeSH) [17], and the Gene Ontology (GO) [18]. Two approaches use metadata, such as authors [15] and Journal Descriptor Indexing [13]. Most of the *unsupervised *approaches so far were evaluated outside the biomedical domain [19-25], with the exception of [26], who used relations between terms given by the UMLS for unsupervised WSD of medical documents and achieved 74% precision and 49% recall. Another approach that uses the UMLS as background knowledge for WSD is that of [27], who compared the results from a naive Bayes classifier and other algorithms (decision tree, neural network) to conclude that different senses in the UMLS could contribute to inaccuracies in the gold standard used for training, leading to varied performance of the WSD techniques. Another approach by [24] is based on a graph model representing words and relationships (co-occurrences) between them and uses WordNet [28] for assigning labels.

Interestingly, most of the above approaches consider the background knowledge sources as terminologies, without taking into account the taxonomic structure or the terms' semantic similarity [29-36]. Here, we fill this gap by systematically comparing three approaches using ontologies with inference and semantic similarity and the use of metadata to solve the problem of WSD for ontological terms. The goal is to establish how the use of ontologies and metadata can improve results.

In previous work [37], we proposed and evaluated two approaches to the WSD problem, namely term co-occurrences in PubMed abstracts and document clustering. We proposed a methodology for finding whether an article in an automatically annotated database is likely to be true or false with respect to the biological meaning and constructed a co-occurrence graph of GO terms based on Gene Ontology Annotations (GOA) [38]. In the present article we extend this approach (called 'Term Cooc') in the following ways: first, we additionally disambiguate MeSH terms and use a larger training corpus to get the co-occurrence scores, since there exist ~16, 400, 000 documents to which experts have assigned MeSH terms. Second, we make use of the hierarchy structure in both GO and MeSH (given an ambiguous term *α*, the co-occurrence of *α *with a term *β *should not be lower than *α*'s co-occurrence with any of *β*'s descendants, 'Inferred Cooc'), whereas before we used term co-occurrence without any inference. We therefore investigate how two different hierarchies influence the performance of disambiguation. Third, we combine our graph-based decision function with a support vector machine, arranged in a co-training scheme, to learn and improve models without any labelled data. Finally, we test the disambiguation performance in new larger benchmark datasets of varying curation quality. The 'Term Cooc' approach is similar to Dorow's approach [24], with the difference that we construct the co-occurrence graph based on GOA and MeSH, which are manually annotated datasets. Therefore, our graphs contain only relations (edges) between terms (nodes) that are semantically meaningful in the context of an article. Dorow's graph contains all the nouns that co-occur with one another, but in the case of the biological context, we are interested only in a local subgraph of Dorow's graph (i.e. 'development' only in the biomedical sense). Another difference is that we use established knowledge in GO and MeSH to draw the nodes and in the different configurations of our method we use a support vector machine and/or incorporate the term relationships in GO and MeSH.

We introduce two more methods for disambiguation, differing from the co-occurrences approach in terms of automation and background knowledge required. The 'Closest Sense' approach computes similarities between the senses of the ambiguous term, the senses of its neighbours (co-occurring terms) and the type of relations that could occur between them. 'Closest Sense' (CS) uses the UMLS semantic network as background knowledge, like [26], who rely on the context of the ambiguous term in order to compute a score for each sense candidate. This score consists of the number of terms in the document which are related, in the UMLS, with the different senses of the ambiguous term. In comparison to the CS method, this approach is different in two main points: (i) it does not take advantage of the hierarchies of concepts and relations in the UMLS and (ii) it ignores terms which co-occur with the ambiguous term in the same context but do not have a direct link with it in the UMLS. The 'MetaData' method uses maximum entropy for modelling the behaviour of occurrence of contextual terms and phrases in text together with a potentially ambiguous term. The features selected are *n*-tuples of word stems and metadata such as the journal and document title. The method requires a set of labelled documents for each term to be disambiguated. We evaluate and compare the three strategies for WSD, starting from the unsupervised/automated to the least automated one. The comparison includes each method's requirements and limitations in terms of training data and automation, the behaviour of the methods during the use of different taxonomies (GO/MeSH/UMLS) as well as comparison against a classical stem co-occurrence approach. We additionally make the benchmark datasets created for the purpose of disambiguation available, since the collection process is time-consuming and labour-intensive. These include 2600 manually curated documents of high/medium curation quality for 7 selected GO and MeSH terms.

## Methods

### Terminology and classification of approaches

The types of relations between terms in the Gene Ontology (GO), the Medical Subject Headings (MeSH) and the Unified Medical Language System (UMLS) semantic network make them completely different knowledge sources [39]. GO has a simple structure in the form of a directed acyclic graph and GO terms are interconnected via *is_a *and *part_of *relations. The semantics of relations used in MeSH make it a terminology rather than an ontology. Terms in MeSH are related through *A narrower than B *relations, giving users who are interested in *B*s the option to look at *A*s. The UMLS is considered to be a terminology integration system comprising over 130 biomedical vocabularies and relations like subClassOf or subPropertyOf between terms. Therefore, it is located in the space between a structured terminology and an ontology. The most popular semantic web formalisms for representing taxonomies, ontologies and terminologies in general are the Resource Description Framework (RDF) and the Web Ontology Language (OWL), with OWL being more suitable for ontologies and RDF sufficient for terminologies. The Simple Knowledge Organization Systems (SKOS) is an area of work developing specifications and standards to support the use of knowledge organisation systems (KOS) such as thesauri, classification schemes, subject heading systems and taxonomies within the framework of the Semantic Web. SKOS provides a standard way to represent knowledge organisation systems using RDF. Lately, there have also been provided OWL translations of GO and MeSH by the responsible consortia.

We designed, implemented, and evaluated three WSD methods that we refer to as: *Closest Sense *(CS), *Term Cooc *(TC), and *MetaData *(MD). These differ as follows:

#### Background knowledge

Closest Sense (CS) uses the UMLS semantic network; it represents an abstract as a list of UMLS terms occurring in the abstract. Term Cooc (TC) uses co-occurrences of terms in GO and MeSH, built from a curated dataset; it represents a document abstract as a list of GO and MeSH terms occurring in the abstract. The MetaData method (MD) uses metadata about the journal and title; it represents a document abstract as *n*-tuples of word stems and metadata.

#### Classification

CS uses shortest semantic distance of co-occurrences to sense. TC uses SVMs and co-occurrences from a training dataset for finding boundary between senses. MD uses the maximum entropy to model the behavior of the co-occurrence of contextual words and metadata with the ambiguous term.

**Figure 1:**
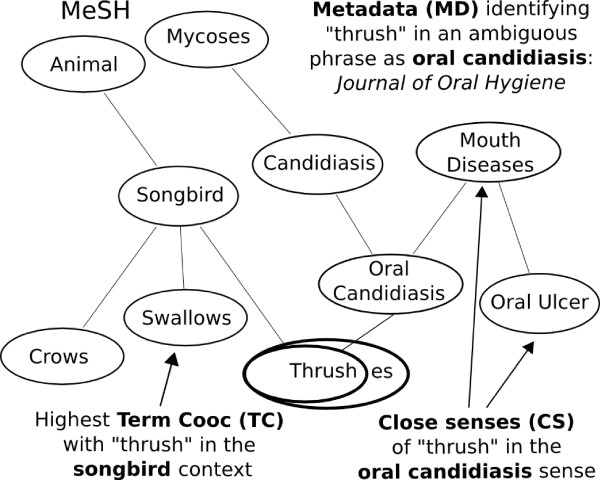
**Three disambiguation approaches for one term**. *Thrush *is an ambiguous term, as its senses include *songbird *or *oral candidiasis*. This figure shows the possibilities for disambiguating 'thrush'. Solid edges are **is_a **relationships.

Figure 1 gives an overview of the disambiguation performed by the three methods. 'Thrush' can refer to a mouth disease (oral candidiasis) or to a songbird (e.g. thrush nightingale). The CS method examines what appears in the same sentence and/or paragraph (e.g. 'mouth diseases' or 'oral ulcer') and then computes a similarity based on semantic distances to 'songbird' and 'oral candidiasis' in the UMLS semantic network, with the highest similarity determining the result. The TC method examines what appears in the same abstract (e.g. 'swallows') and then considers all known co-occurrences between taxonomy terms in the training corpus. The value of the highest co-occurrence determines the result, e.g. 'swallows' would have relatively high co-occurrence with 'thrush' songbird. The MD method uses metadata for the document and then decides based on what was previously learned about this metadata from training examples. If, for example, the article comes from the Journal of Oral Hygiene, then it is more likely that 'thrush' refers to 'oral candidiasis'.

### Closest Sense method (CS)

This WSD approach was initially used to address the ambiguity problems in the MeatAnnot system [40]. The main idea of the approach is the following: given a set of different senses of the ambiguous term, the co-occurring terms in the same text and the hierarchy where they belong (including the different types of relations), decide which sense is true based on the (shortest) distance to the senses of the co-occuring terms. To clarify this, we can have the following sentence as an example: *'I also tracked lipid profiles, HBA1C, blood pressure, body mass index, hostility and nicotine use'*. The term 'blood pressure' can have three senses, namely 'organism function', 'diagnostic procedure' and 'laboratory or test result'. The senses of the co-occurring terms are 'laboratory procedure' (lipid profile), 'gene or genome' (HBA1C), 'diagnostic procedure' (body mass index), 'mental process' (hostility) and 'organic chemical' (nicotine). The sense of 'diagnostic procedure' for blood pressure is in average closer to the senses of the co-occurring terms than the other candidate senses. For the example in Figure 1, the CS method determines the meaning of 'thrush' by examining what appears in the same sentence and/or paragraph (e.g. 'mouth diseases' or 'oral ulcer') and then computing a similarity based on semantic distances to 'songbird' and 'oral candidiasis' in the UMLS hierarchy; the highest similarity determines the result. Intuitively, with semantic distances, two senses are close if there exists a possibility to use them in a concise annotation graph.

#### Algorithm

The 'Closest Sense' algorithm takes as input: (*i*) the ambiguous term *τ*, (*ii*) the vector *V*_*τ *_of different senses of *τ*, (*iii*) the vector *VC*_*τ *_of senses found in the context (sentence and/or paragraph containing the ambiguous term *τ*), and (*iv*) the UMLS semantic network.

First, the disambiguator builds a vector *VC*_*τ *_of senses describing the context of the ambiguous term *τ*.

This vector includes the senses of terms that are neighbours of *τ*. Then, it computes the similarity between each sense in vectors *V*_*τ *_and *VC*_*τ*_.

The resulting similarity is the average of similarities between senses in the two vectors. Finally, the sense in *V*_*τ *_that has the highest average similarity to *VC*_*τ *_is proposed as the best for *τ*.

#### Semantic distances

The distance metrics used to find the correct sense are the *subsumption distance *and the *subtype-aware signature distance*.

The *subsumption distance *is the length of the shortest path between two *senses *in the hierarchy of senses, where the length of an individual subsumption link gets exponentially smaller with the depth of the senses it links in the hierarchy.

The *subtype-aware signature distance *is the length of the shortest path between two *concepts/terms *through the graph formed by the property types with their range links and domain links. With this new semantic distance we merge signature and hierarchies graphs. The main idea is to find a path between two concepts/terms by using the ontology structure (subClassOf relations between terms, subPropertyOf relations between properties) and the signature of relations (domain and range). The subtype-aware signature consists of relations in the hierarchy (subClassOf, subPropertyOf) additional to the common signature (domain and range of a property). It is *aware *of the properties of a term (signature), the position of the term in the hierarchy (subClassOf relations) and the hierarchy of the properties (subPropertyOf relations).

**Figure 2:**
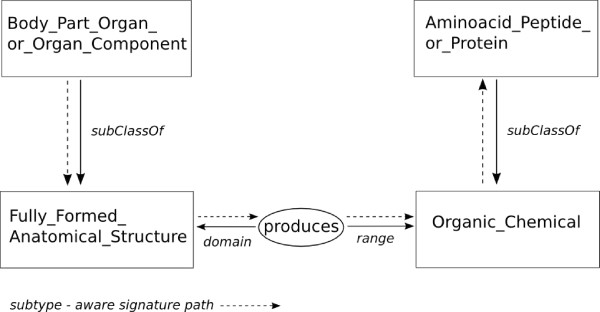
**Subtype-aware signature calculation**. The figure shows the path between the UMLS terms 'Body_Part_Organ_or_Organ_Component' and 'Amino_Acid_Peptide_or_Protein'. The edges describe relations between entities (in our case, the subtype-aware-signature and its sub-properties) and nodes consist of classes and relations of the ontology. 'Body_Part_Organ_or_Organ_Component' is a subsumption of 'Fully_Formed_Anatomical_Structure', which belongs to the signature of the relation 'produces'. This relation has as range 'Organic_Chemical' which is a super-class of 'Amino_Acid_Peptide_or_Protein'. The length of this path is 4.

Figure 2 provides an example of the *subtype-aware *signature distance calculation between two terms in the UMLS semantic network. 'Body_Part_Organ_or_Organ_Component' is a subClassOf 'Fully_Formed_Anatomical_Structure', which belongs to the signature of the relation 'produces'. This relation has as range 'Organic_Chemical' which is a superClassOf 'Amino_Acid_Peptide_or_Protein'. The *optimized distance *is a combination of the *subsumption distance *and the *signature distance*, parameterized with three *optimal weights*, *w*_*sig *_for signatures, *w*_*subclass *_for class subsumption links and *w*_*subprop *_for property subsumption links. From a first experiment on the UMLS WSD test collection [4] where we tested different weights starting from a distance favouring the class subsumption relation to a distance favouring the signature relation, we ended up in the following optimal weights giving the best accuracy: *w*_*sig *_= 0.4, *w*_*subclass *_= 0.2 and *w*_*subprop *_= 0.4.

### Term Cooc method (TC)

The Term Cooc method relies on selecting the term that most frequently co-occurs with the ambiguous term in the training corpus. It selects the highest co-occurring term with the ambiguous term for defining the given sense as true or false. In order to formalize the notion of term co-occurrences (GO or MeSH), we consider pairs of GO/MeSH terms that appear in the same abstract and we represent all such pairs of terms in a manually annotated GOA or MeSH co-occurrence graph (see training with co-occurrence graphs subsection below). Each node in the co-occurrence graph represents a GO or MeSH manual annotation. An edge between nodes *α *and *β *represents a real number, the log-odds score, representing the frequency *log *– *odds*(*α*, *β*) of the terms' *α *and *β *co-occurrence over all articles, weighted by their total number of occurrences.

For the example in Figure 1, the TC method determines the meaning of 'thrush' by examining what appears in the same abstract (e.g. 'swallows') and then considering all known co-occurrences between ontology terms in a training corpus; the value of the highest co-occurrence determines the result, e.g. 'swallows' would have relatively high co-occurrence with 'thrush' songbird.

#### Algorithm

First, we use a simple threshold considering how close to an ambiguous term the highest co-occurring term (of the ones in the article) is; if below a user-defined threshold *θ*, the ambiguous term is negative, else it is positive with respect to the term. Second, we use Support Vector Machines (SVMs) trained on all tokens of a text [41].

The method first runs a binary SVM against a set of articles ordered by maximum co-occurrence with the ambiguous term. The highest and lowest 10% of articles in the set are labelled as positive and negative; then the SVM is trained on lower 10% (article with least co-occurring term with the ambiguous term) and upper 10% (article with highest co-occuring term with the ambiguous term). After the initial convergence is achieved, the error (wrongly classified vectors) will be low, likely near 0. The algorithm next improves this result by iteratively re-classifying the remaining articles that have less extreme co-occurrences with the ambiguous term, one-by-one, followed by re-training the SVM on the newly relabelled data set. This continues until no more articles are left. The steps are:

1. *Set S *= Order articles based on their highest co-occurring GO/MeSH annotation (from the co-occurrence graph) with the ambiguous term.

2. *T *= lowest and highest 10% of *S*; label *T *as negative and positive; train SVM with *T*; remove *T *from *S*.

3. For *s *∈ *S*: move *s *to *T*; classify *s*; re-train SVM with *T*.

#### Training with co-occurrence graphs

These graphs are used in the TC method for *training*. For WSD of GO terms, the co-occurrence graph derived from the Gene Ontology Annotations (GOA) [38], and for the MeSH terms from the Medical Subject Headings (MeSH) [17]. GOA represents articles manually annotated with GO terms and consists of ~34,000 articles. There exist ~16,400,000 documents to which experts have assigned MeSH terms. We found co-occurring terms in the GOA and MeSH annotated articles and built a co-occurrence graph representing how frequently pairs of GO or MeSH terms co-occur. Nodes represent annotations and edges represent the frequency of co-occurrence of two annotations in the same article, normalized based on each GOA/MeSH annotation's individual occurrence frequency in the specific corpus.

#### Training with inferred co-occurrences

We extend the co-occurrences in a hierarchical fashion to ensure that given a GOA-derived co-occurrence between a pair of terms, *GOAcooc*(*α*, *β*), the ancestors of *α *and *β *in the ontology are updated with the co-occurrence such that only the maximum co-occurrence is kept. This is important given the few annotations in GOA and the is_a relationships between GO terms, since ancestors inherit the co-occurrences of their children.

With the inferred co-occurrences, given an ambiguous term *α*, the co-occurrence of *α *with a term *β *will not be lower than *α*'s co-occurrence with any of *β*'s descendants.

### MetaData method (MD)

As an alternative method for WSD we use a maximum entropy approach as described in [42,43]. Maximum entropy models have been successfully used in tasks like part of speech tagging, sentence detection, prepositional phrase attachment, and named entity recognition.

For the example in Figure 1, the MD method determines the meaning of 'thrush' by using metadata for the document and then deciding based on what was previously learned about this metadata from training examples. The metadata used are *n*-tuples of word stems from different scopes, namely the paper title, the sentence including the ambiguous term or the whole abstract, the journal title as well as the publication period, since some topics can be popular in different decades. The occurrence of contextual words and phrases in a text together with a potentially ambiguous term can be seen as a random process. Maximum entropy modelling aims at modelling the behavior of this random process. Provided a large amount of training examples, the algorithm automatically extracts a set of relationships inherent in the examples, and then combines these rules into a model of the data that is both accurate and compact.

#### Algorithm

The *training *and *test *data in our case are sentences containing the potentially ambiguous term flagged with the sense. Each training example, one sentence each, is represented as a set of features. An implementation of the Porter stemmer is used [44] and as features we select *n*-tuples of word stems and meta information of the document, such as the journal and title words and the publication period (10 years ranges).

The *implementation *[45] takes a series of events to train a model. Each event is a configuration of binary relations associated with a label. The resulting model is applied to an unknown configuration of binary relations. The result is the predicted probability for the previously trained outcomes. MeSH terms already assigned to the articles are excluded, for the performance evaluation to be independent of them.

Given the abstract of a scientific article and the ambiguous term, the steps followed are:

1. extract binary features (*n*-tuples of word stems from different scopes – title, sentence, entire abstract -, publication period, journal title)

2. get scalar product of feature vector and model (vector based on training)

3. the result is the probabilities for predefined outcomes (in this case True or False)

4. if above a threshold 0.5, the term is True, else False.

As an illustrating example of the features extracted, articles mentioning 'signal transduction', 'kinase', 'embryo', 'neuron' or 'stage' are more likely to refer to 'multicellular organismal development' than to another sense, such as development of an algorithm or a disease in an organism. Articles mentioning 'anxiety', 'behaviour', 'memory', 'social' and 'fear' are more likely to refer to 'psychological inhibition', instead of 'enzyme inhibition'.

## Experimental setup

### Classification task and limitations

The disambiguation performed here is mainly a classification task; it represents the prediction whether an annotation is positive or negative with respect to the GO/MeSH sense. We do not assign one of the numerous different senses to a term, but instead a positive or negative label to it, when it corresponds to the GO/MeSH sense or not, respectively. We do not handle acronym ambiguity separately. However, in cases where an acronym belongs to an ontology term label (e.g. FA for GO term 'Fanconi Anaemia' vs 'Fatty Acids', AMP as of MeSH term 'Adenosine Monophosphate' vs 'Antimicrobial Peptides', etc.), this is disambiguated in the same way as all ontology term labels.

As mentioned in the introduction, some disambiguation tasks are easier than others; 'bank' the building and the 'BANK' gene will appear in completely different context, whereas the 'BANK' gene, protein or mRNA are even likely to appear in the same article abstract, making the disambiguation task often difficult even for a domain expert. 'Transport by air' or 'patient transport' will be easier to distinguish from the GO sense of transport, but 'transport of virus cultures' will appear in a closer molecular biology context. Distinguishing between 'transport', 'RNA transport', 'tRNA transport' or 'ion transport' can become less difficult by using the hierarchical information in the ontology (e.g. exploiting subClassOf/subPropertyOf relations between ontology terms). Some terms are also easier to disambiguate in the same task, depending on the number of their different senses (see Table 2) and the distance between them, the way they appear in text (e.g. some can be easily distinguished with the help of regular expressions) and the number of tokens they consist of (one-token terms are usually more ambiguous as they are more likely to correspond to common English).

**Table 2:** Ambiguous terms and their senses in the WSD datasets collected.

	**Term**	**Senses**
GO	Development	biological process of maturation (GO); development of a syndrome/disease/treatment; cataract development; colony development; development of a method; staff/economic development; software/algorithm development
	Spindle	mitotic spindle (GO); sleep spindles; muscle spindle; spindle-shaped cells
	Nucleus	cell nucleus (GO); body structure (UMLS, subthalamic/cochlear/caudate nucleus); aromatic nucleus
	Transport	directed movement of substances into/out of/within/between cells (GO); patient transport (UMLS); transport by air; transport of virus cultures; maternal transport

MeSH	Thrush	Oral Candidiasis (MeSH); songbird (e.g. thrush nightingale)
	Lead	heavy metal (MeSH); lead measurement (UMLS); to result in
	Inhibition	psychological/behavioral inhibition (MeSH); metabolic inhibition (UMLS); % inhibition (SNOMED)

Examples of the senses (in and out of the taxonomies) per ambiguous term included in the benchmark dataset collected.

The ambiguous terms examined are the GO terms 'Development' (GO:0007275), 'Spindle' (GO:0005819), 'Nucleus' (GO:0005634) and 'Transport' (GO:0006810) and the MeSH terms 'Thrush' (D002180), 'Lead'(D007854) and 'Inhibition' (D007266). Most of the different senses of the terms examined (see Table 2) belonged as well to the biomedical domain, making the disambiguation task more difficult (e.g. development of a cell culture, development of a cytopathic effect, maturation-GO development). The limited number of terms examined is due to the labor-intensive process of manual collection of proper benchmark datasets. As mentioned in the introduction, there exist few annotated biomedical corpora for evaluation and depending on the task, researchers need to collect their own gold standard datasets. We collected datasets for a list of ambiguous terms based on the amount of true/false data available and the frequency of occurrence in PubMed (2600 manually curated documents of high/medium curation quality for 7 selected GO and MeSH terms). We aimed at keeping the ratio of true/false abstracts close to 1, giving a 50% chance to each appearance of the term to be true or false with respect to the GO/MeSH sense (although the ratios in Medline will be different per term). We first examined the UMLS WSD collection [4] for ambiguous GO/MeSH terms and data availability and later a list of common False Positive terms based on manual curations in GoPubMed (terms that were often falsely annotated by GoPubMed as GO/MeSH terms and curators disagreed with the automatic annotation). From the UMLS WSD collection we selected terms that were GO/MeSH terms and the senses provided were distant to each other, i.e. in the case of 'lead', the two senses with short semantic distance (compound; laboratory procedure of lead measurement) were considered as one, as they both are about the compound. A semantically more distant sense is that of the verb to lead/result in. Regarding the false/positive ratio limitation/criterion, for some terms this was not satisfied, not allowing the inclusion into the evaluation dataset. For example, for 'transport' the UMLS WSD collection contained 93 abstracts classified as sense1 (True for GO sense) and only 7 as other (curators in this collection had 3 options: sense1, sense2 or other, here sense1 as the biological transport and sense2 as patient transport). We therefore needed to manually collect False examples containing other senses for a balanced corpus.

Another question was whether the definition of the negative datasets would influence the results. To test this, we defined a more general negative dataset by completely randomly choosing articles. Defining a random set as a negative set is common practice, e.g. in predicting protein-protein interactions. Obviously, the random negative dataset is very different from the positive dataset, since most likely it does not contain any of the negative senses at all, but is just the bias for the "average" paper. Results showed a decrease of ~7% in the performance of the methods.

This argument can be turned around. While our initial negative dataset was carefully and manually chosen, it could be further improved by letting its composition of other senses reflect the distribution of use of these senses in PubMed as a whole. However, achieving this ideal would require annotating all articles with the term in the whole of PubMed with the senses. Given that PubMed has for example more than 1 million articles on development, this cannot be easily accomplished.

However, the composition of negative senses in our negative dataset aims to reflect the composition of negative senses in PubMed as well as possible through the query and annotation strategy, that was pursued. Since we needed to include every possible sense of the ambiguous terms, the queries formed were such that could collect representative abstracts for each sense, a process that was manual and time-consuming. The collection of the positive examples was easier, since there was one sense (with respect to the taxonomy) and also more frequent in PubMed, therefore the term itself or one of its synonyms were enough to be put in the query to PubMed. The collection of negative examples was as expected harder, since they were not frequent in PubMed and we needed to include enough examples of every possible sense. Most of the queries used for this included the ambiguous term or synonyms of it and keywords that were often in the context, based on personal experience from previous curation of automatic annotations in GoPubMed. For example, for 'development', the queries used were 'development AND staff', 'development AND algorithm', 'development AND software', 'development AND treatment', 'development AND method', etc. For 'thrush', since we could only locate one negative sense, we used queries such as 'thrush nightingale', 'thrush AND songbird', 'mountain thrush', etc.

The other aspect is the question of size composition of positive and negative. We chose roughly 50% positive and 50% negative. This basically means that the a priori likelihood is 50% for the corect sense. If, instead, we aim to identify each sense correctly, the following problem arises: assume there are ten senses, i.e. 1 positive and 9 negative. Then the a priori probability for the classification would be 10% and then a simple strategy would be to always vote negative.

Overall, the approach pursued (manual selection of negative senses, roughly covering the common negative senses) plus equally weighted positive and negative datasets is a suitable approach for evaluation.

### Datasets

We collected three different benchmark datasets (see Table 3) to evaluate the performance of the three methods. They differ in quality and quantity, depending on their collection process (manual by one curator, directed manual by several curators, mainly automatic). The common reference dataset between the three methods is the *manually annotated by a domain expert *one:

**Table 3:** Benchmark datasets for WSD.

	**Term**	**Manual (expert)**		**Manual (non-experts)**		**Semi-automatic**	
		False	True	False	True	False	True
GO	Development	98	111	271	56	2296	715
	Spindle	50	48	70	48	519	599
	Nucleus	99	100	25	61	131	1336
	Transport	102	91	102	56	1043	699

MeSH	Thrush	17	83	45	7	35	1131
	Lead	71	27	202	22	1564	735
	Inhibition	98	100	454	79	5247	553

The above datasets contain *manually *collected PubMed articles by one expert (high quality/low quantity), *manually *curated articles by a group of non-experts (medium quality/medium quantity) and *semi-automatically *collected articles (low quality/high quantity). See Datasets section for details.

#### High quality, low quantity corpus

this corpus consists of ~100 true and 100 false example documents (abstracts) per ambiguous term. For the ambiguous GO terms examined and the MeSH term thrush we collected both true and false examples *manually*. True examples are abstracts that discuss, for instance, 'Development', in the sense specified by GO. False examples also contained the ambiguous term, but in other senses, closer or not (see Table 2). For the ambiguous MeSH terms 'Lead' and 'Inhibition (psychology)', the test set originated from the UMLS WSD corpus [4]. These two were the only terms depicting MeSH terms. All other terms in the UMLS WSD (such as growth, repair and reduction) were only found in GO or MeSH as substrings and would thus not be contained in either co-occurrence graph as single nodes.

#### Medium quality, medium quantity corpus

this corpus consists of documents for which the annotation has been *manually confirmed *by a group of expert and non-expert curators. We asked colleagues to confirm or reject the automatic annotations (for GO and MeSH terms) provided by GoPubMed for a collection of article abstracts. This collection has been mainly automatically created, as described next (low quality, high quantity corpus). For each of the automatic annotations, the curators could select among three options: *a*. true and important for the context of the publication, *b*. of minor importance/relevance and *c*. false annotation. The curation tool is available via GoPubMed [46].

#### Low quality, high quantity corpus

this corpus was created mainly *automatically*. We implemented similarity-based clustering of abstracts with literal occurrence of the ambiguous terms. Each abstract was matched to its nearest abstract, conceptualized as a directed edge from the former to the latter. Then every connected component was considered as a cluster. From an initial manual evaluation of the clustering results, clusters of size > 60 were consistent enough, meaning that articles in such clusters were referring to one sense of the ambiguous term in 72–95% of the cases. Each cluster's abstracts were input into a system developed in-house (also used in [47]) that generated a list of terms describing each cluster based on term frequency inverse document frequency (TFIDF). The top 20 terms of the list were later evaluated by an expert which labelled the clustered articles as true or false for the respective GO/MeSH term. The above facilitated and accelerated the dataset collection process without any significant loss in data quality (compared to the gain of data quantity for benchmarking).

### Experiment

For evaluation and comparison purpose, each method's performance was tested (in terms of precision, recall and specificity) on the high quality/low quantity dataset (see CS1-2, TC1-4 and MD1-3 in Table 4 and Additional file 2 for specificity and detailed results per method). We also applied classical stem co-occurrence analysis as a baseline on the same dataset; this consisted of basic maximum entropy modelling on stems without any use of metadata or hierarchical information (see bME in Table 4). We additionally tested each method's performance separately with different test datasets. For the 'Term Cooc' method (TC), the performance of co-occurrences of GO/MeSH terms and inferred co-occurrences of GO/MeSH terms (each one of the variants combined -or not- with Support Vector Machines) was tested in the three benchmark datasets described earlier, in order to evaluate the method in larger (but of lower quality) datasets, since it has been shown that sample size, sense distribution and degree of difficulty impact on the classification task [48]. Input to this method were the automatic annotations per article provided by GoPubMed (GO/MeSH terms and MeSH hand annotation) and the respective co-occurrence graph. As a side experiment, we tested the TC method for the disambiguation of MeSH terms without including the MeSH hand annotations in the automatic annotations provided by GoPubMed, to estimate how the quality of the input influences the quality of the results. For the 'Closest Sense' method (CS), input was the UMLS semantic network and the article abstracts. This method was additionally tested on the WSD Test Collection [4]. For the 'MetaData' method (MD), we used the three different datasets for training and testing. The high quality dataset was used in an initial experiment (MD1) as training and testing dataset, in a 5-fold cross validation. Then the medium quality and low quality datasets were separately used as training sets, with testing of the method on the high quality dataset (MD2 and MD3, respectively).

**Table 4:** Results (% f-measure) for the baseline (bME) and the three methods (Closest Sense, Term Cooc, MetaData) for 7 ambiguous terms, tested on a high quality/low quantity corpus (manually annotated by expert).

**Term**	**CS1**	**CS2**	**TC1**	**TC2**	**TC3**	**TC4**	**bME**	**MD1**	**MD2**	**MD3**	**Avg**
Development	87	86	74	71	57	79	90	96	80	80	80
Spindle	70	79	90	80	95	98	98	100	77	78	87
Nucleus	89	94	81	78	75	95	97	99	91	77	88
Transport	83	71	90	89	88	94	89	98	91	88	88

Thrush	88	94	87	82	78	81	82	94	94	58	84
Lead	36	53	89	49	93	81	85	85	36	14	62
Inhibition	66	84	77	62	85	58	92	100	95	97	82

Avg	74	80	84	73	82	84	90	96	81	70	81

CS1 column contains the results (% *f*-measure) for the Closest Sense (CS) approach with the use of the classic distance (only subsumption). CS2 column contains the results for the CS approach with the use of the optimized signature together with the subsumption distance. TC1 and TC2 contain the results of the Term Cooc (TC) approach, when the co-occurrences or the inferred co-occurrences are used, respectively. TC3 contains the results for the TC approach with co-occurrences and support vector machines, and TC4 when inferred co-occurrences and SVMs are used. bME column contains the results for the baseline method (classical Maximum Entropy modelling of stems without metadata or hierarchical information), trained and tested on the high quality corpus in a 5-fold cross validation. MD1 is for the MetaData approach, trained and tested on the high quality corpus in a 5-fold cross validation. MD2 is trained on the medium quality/quantity corpus and tested on the high quality one. MD3 was trained on the low quality/high quantity corpus and tested on the high quality corpus. Some terms (spindle, nucleus, transport) are easier to disambiguate than others (development, lead). Overall, all methods perform well between 73–96% *f*-measure (*f*-measure, *F*, is the weighted harmonic mean of precision, *P *and recall, *R*: *F *= 2 × *P *× *R*/(*P *+ *R*)).

## Results

The performance of the three disambiguation approaches (CS, TC, MD) and the baseline (bME) was tested on a common high quality/low quantity dataset. The overall results of this comparison are shown in Table 4 (detailed results per method are given in Additional file 2). All methods perform well between 73–96% average *f*-measure. In particular, the MetaData (MD1-3) approach is the best one: when trained on high quality data (MD1), it achieves 96% *f*-measure. When the metadata are not used (baseline method, bME) the accuracy falls to 90%. The Term Cooc (TC1-4) method follows with 81% and the Closest Sense (CS) approach with 77% (80% for the optimized signature together with the subsumption distance, in CS2). All methods present low *f*-measure for 'development' and 'lead' (79% and 60% in average). The best results (in average for all methods) are obtained for GO terms 'transport', 'nucleus' and 'spindle' (88%, 87% and 85% respectively).

As far as the Closest Sense approach is concerned, there is a clear improvement in the results (from CS1 to CS2) with the use of the optimized signature together with the subsumption distance. For the Term Cooc approach, when the inferred co-occurrences are taken into account (the scores are propagated to the parents of the terms, from TC1 to TC2) in the case of the GO terms the results remain the same, whereas in the case of the MeSH terms the results are worse, mostly in terms of recall (see Additional file 2). For GO terms, the results are best when inferred co-occurrences are combined with SVMs (TC4, 79–98% *f*-measure), whereas for MeSH terms, the best *f*-measure (79–93%) is achieved when co-occurrences with SVMs are used, without the inferred co-occurrences (TC3). This difference can be explained by the different structure of the two hierarchies. GO can be described as "tall and thin" (few children per node, many levels, with maximum number of levels 19), whereas MeSH is "short and fat" (many children per node, not many levels, with a maximum of 9 for the version of 2007). Additionally, the relations between terms in MeSH are not exact is_a relations, but rather is_related_to. Therefore, propagating the term co-occurrences in MeSH does not improve the results, since it does not necessarily mean that annotating with term *MeSH*_*X *_also means all of *X*'s ancestors. On the contrary, in GO this is more likely to hold.

The MetaData method gives – as expected – the best results. When the method is trained and tested on the same high quality test (with a 5-fold cross validation, see MD1), it results in an average *f*-measure of 96%. When trained on the medium quality (MD2) and low quality (MD3) corpora and tested against the high quality corpus, the *f*-measure decreases into 81% and 70%, respectively, which are nonetheless high, compared to the quality of the training sets. The high performance of the MetaData approach is mainly due to the use of metadata as the title of the abstract and the journal. For example, for the terms 'inhibition' and 'spindle' it achieves 100% *f*-measure and for 'nucleus' 99%. The true sense of inhibition for MeSH is psychological inhibition, which is easier to disambiguate, since it will mostly appear in psychology/psychiatry journals. The same applies for 'spindle', which will mostly occur in cell biology and cell division/cycle journals.

We additionally tested each method's performance separately with different test datasets (data not shown here). The 'Closest Sense' method was also tested on the NLM UMLS WSD Collection [4] to compare four versions of semantic distance computation in order to disambiguate term mapping to the UMLS semantic network. The experiment showed that the use of the ontology definition can improve significantly the precision. Over the 22 ambiguous terms examined, the overall average precision was 83%.

For the 'Term Cooc' method, the performance of the different variants (co-occurrences +/- inferred co-occurrences +/- SVMs) was tested in the three benchmark datasets described earlier (see Datasets section and Additional file 1), in order to evaluate the method in larger (but of lower quality) datasets.

Testing the method from the highest towards the lowest quality (but higher quantity), the *f*-measure decreases only by 3–10%, indicating a consistent behavior of the method. As a side experiment, we tested the 'Term Cooc' method for the disambiguation of MeSH terms without including the MeSH hand annotations in the automatic annotations provided by GoPubMed, to estimate how the quality of the input influences the quality of the results. As expected, the results decreased dramatically (~46%), indicating that the MeSH hand annotations provided per article are important for the disambiguation (see Additional file 2).

## Discussion

Overall, the MD method gave the highest *f*-measures among all methods. The results became worse for the medium and high quantity datasets, since these were of lower quality in terms of correctness. The MD approach's consistency with giving the highest results is due to integrating metadata, such as journal and title, which are representative of the true meaning of an ambiguous term. The MD approach needs plenty of labelled data for training.

When comparing the results of the TC and CS methods to the baseline method (bME) that performs only maximum entropy modelling of stems (without use of metadata), bME still gives better results, but this is due to the available training data of high quality. The disadvantage of MD and bME compared to TC and CS is the need of high quality training data.

The MD approach is less scalable in terms of storage demands as the number of articles increases, while the CS and TC approaches have constant storage demands (ontology and a co-occurrence graph). In the TC method the SVMs increase the results up to 98%. The TC method requires an ontology and co-occurrence graphs. The origin of this graph should be a manually curated data source, in our case GOA and MeSH. The quality of the graph will heavily depend on its origin and quality of the data.

**Figure 3:**
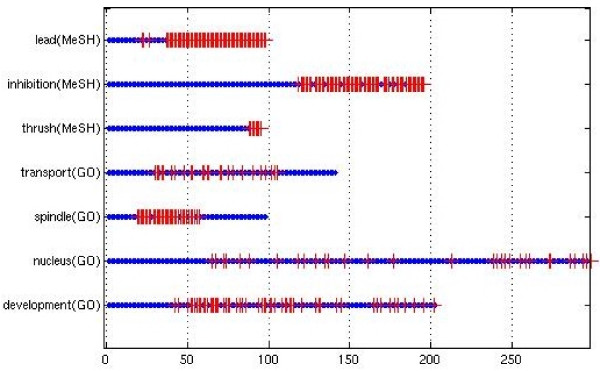
**Term Cooc classification over time**. The *x*-axis is the TC classification over time. Left-most articles are classified early, since they have the highest or lowest co-occurrences with the ambiguous term. Red crosses are errors or wrong predictions. Almost none of the early classified articles are errors.

The inferred co-occurrences improve the results for GO, while for MeSH they get worse. This is due to the different structures of the two semantic hierarchies; the ancestors of an applicable GO term are more likely to also be applicable to the same article, because of GO's structure that is "tall and thin". But MeSH's structure is "short and fat" and is not always a thesaurus; not all of a node's ancestors are also applicable. Moreover, in the TC method the inferred co-occurrences only improve the result if combined with the SVM. This is because the inferred co-occurrences make the extreme co-occurrences with the ambiguous term, which the SVM uses for training, more representative of an ambiguous term's true meaning. Figure 3 shows that the most extreme co-occurrences with the ambiguous term are most likely to be classified correctly, since the inferred co-occurrences make more precise the highest and lowest co-occurrences with an ambiguous term. The middle co-occurrences are not necessarily made more precise with inferred co-occurrences. That is why inferred co-occurrences help with the (initial) SVM training; while later on for middle co-occurrences the errors accumulate.

The CS approach needs only a semantic hierarchy in the form of an ontology, and in this sense is the most automated of the three methods. Moreover, CS gives good results, where the only problematic term is 'lead'. However, CS is sensitive to the design of the ontology or subdomain of UMLS used, which reflects the view of the designers. As shown by the accuracy of [9,13], UMLS may not be the best choice to be used as background knowledge as the different parts of the hierarchy are modelled differently (MeSH, GO, SNOMED, etc.), resulting in different granularity. Different groups of people design ontologies differently; the various subdomains of an ontology will reflect the designers' views respecting depth, number of nodes, and structure. Therefore, the subdomains of the ontology influence the performance of the CS method, and the design rationale of the ontology may be ultimately responsible for performance differences on various terms. For example, 'nucleus' is a subtree root in both GO and SNOMED (anatomical structures); in GO there are 2000 descendants of nucleus, while in SNOMED 10.

## Conclusion

Based on the results, metadata and training data of high quality seem to be the key point for the increase of the accuracy. When such training data are not available – as happens in most of the cases – co-occurrence of ontology/taxonomy/thesaurus terms can provide the way to the right decision. Moreover, the hierarchy of the terms and the subdomain, when consistently modelled, can depict the correct sense of an ambiguous term.

The MD method produced the best results by including metadata in the WSD decision, but it requires high quality training data. The most interesting thing about the TC and CS methods is that they are semi-automated, given a co-occurrence graph or ontology; then the training does not require manual intervention. TC requires well modelled ontologies such as GO, and deteriorates as the structure becomes less rigorous as in MeSH. CS requires large and consistently modelled ontologies, which are two opposing requirements. Thus, for TC and CS the structure of the ontology and subdomain affect the distance metric used and WSD quality. Future work will include identifying ambiguous terms for a certain corpus automatically. For this purpose, we will employ WordNet, clustering, Part of Speech and noun phrase statistics, and expert input.

For TC and CS, we assumed that the other terms in the context are correct and independent of one another; in fact, they could also be ambiguous and therefore false. For CS we will optimize the distance computation and propose other distances, taking into account existing annotation bases and ontology structure.

So far, the disambiguation performed was between the true sense in the hierarchy and all other senses that were considered as false. A possible extension of the methods would be to correctly identify if a sense occurs that is not included in the thesaurus/ontology and possibly add it. The Closest Sense method can potentially do this by setting a threshold. From all distances below this certain threshold, one should be clearly shortest. If not, then this indicates a new sense. The Term Cooc and MetaData approaches could be adjusted to identifying new senses by training each method on each sense and setting a certain threshold. If the sense found is not above the threshold, then this can be a new sense.

## Abbreviations

WSD: Word Sense Disambiguation; GO: Gene Ontology; GOA: Gene Ontology Annotations; MeSH: Medical Subject Headings; UMLS: Unified Medical Language System; SNOMED: Systematized Nomenclature of Medicine; SVM: Support Vector Machine; TFIDF: Term Frequency Inverse Document Frequency; CS: Closest Sense method; TC: Term Cooc method; MD: MetaData method; bME: basic Maximum Entropy (baseline method).

## Authors' contributions

DA and BA developed the Term Cooc method with the assistance of JH. AD implemented the MetaData method and KK and FG the Closest Sense method. TW implemented the terminology extraction method used in data collection. HD assisted in data collection. DA performed the manual collection and evaluation of the benchmark datasets and drafted the manuscript. MS supervised and coordinated the project. All authors have read and accepted the final manuscript.

## Supplementary Material

Additional file 1 **Benchmark datasets used in the experiments.** The three corpora (High quality/Low quantity corpus; Medium quality/Medium quantity corpus; Low quality/High quantity corpus) are given in the form of PubMed identifiers (PMID) for True/False cases for the 7 ambiguous terms examined (GO/MeSH/UMLS identifiers are also given). Click here for file

Additional file 2 **Detailed results for all methods.** Detailed results for all methods given in terms of Precision, Recall, Specificity and F-measure. Additional training and testing of the MetaData method in all three corpora, Closest Sense results with different threshold, Term Cooc results with MeSH text-mined annotations only, Closest Sense results on terms from the WSD Test collection [4]. Click here for file
