# Supplementary material for: Biomedical word sense disambiguation with ontologies and metadata: automation meets accuracy
Source: BMC Bioinformatics. 2009 Jan 21;10:28. doi: 10.1186/1471-2105-10-28 (PMC2663782; doi:10.1186/1471-2105-10-28)
Supplement: Additional file 2 — Detailed results for all methods. Detailed results for all methods given in terms of Precision, Recall, Specificity and F-measure. Additional training and testing of the MetaData method in all three corpora, Closest Sense results with different threshold, Term Cooc results with MeSH text-mined annotations only, Closest Sense results on terms from the WSD Test collection [4]. [file 1471-2105-10-28-S2.pdf]

Biomedical word sense disambiguation with ontologies and metadata: automation meets accuracy

Dimitra Alexopoulou[1†], Bill Andreopoulos[1†], Heiko Dietze[1], Andreas Doms[1†], Fabien Gandon[2], Jörg Hakenberg[1], Khaled Khelif[2†], Michael Schroeder[*1] and Thomas Wächter[1]

[1]Biotechnology Center (BIOTEC), Technische Universität Dresden, 01062, Dresden, Germany
[2]INRIA Sophia Antipolis, 2004 Route des Lucioles, 06902, Sophia Antipolis, France

Email: Dimitra Alexopoulou - dimitra.alexopoulou@biotec.tu-dresden.de; Bill Andreopoulos - williama@biotec.tu-dresden.de; Heiko Dietze - heiko.dietze@biotec.tu-dresden.de; Andreas Doms - andreas.doms@biotec.tu-dresden.de; Fabien Gandon - Fabien.Gandon@sophia.inria.fr; Jörg Hakenberg - joerg.hakenberg@biotec.tu-dresden.de; Khaled Khelif - khaled.khelif@sophia.inria.fr; Michael Schroeder*- ms@biotec.tu-dresden.de; Thomas Wächter - waechter@biotec.tu-dresden.de;

*Corresponding author

Supplementary Tables

Table 6 - High quality / low quantity corpus: Precision / Recall / Specificity / F–measure for the baseline (bME) method on the GO and MeSH test datasets.

5-fold cross validation on the high quality/low quantity corpus.

Term	neg	pos	P	R	S	F
Development	111	98	0.84	0.98	0.81	0.9
Spindle	50	48	1	0.96	0.96	0.98
Nucleus	99	200	0.98	0.96	0.96	0.97
Transport	102	91	1	0.81	0.75	0.89
Thrush	17	80	0.77	0.88	0.87	0.82
Lead	71	27	0.96	0.77	0.71	0.85
Inhibition	98	100	0.93	0.92	0.91	0.92

Table 7 - High quality / low quantity corpus: Precision / Recall / Specificity / F–measure for the MetaData (MD) method on the GO and MeSH test datasets.

5-fold cross validation on the high quality/low quantity corpus.

Term	neg	pos	P	R	S	F
Development	98	111	0.92	1	0.91	0.96
Spindle	50	48	1	1	1	1
Nucleus	99	200	1	0.99	0.99	0.99
Transport	102	91	0.96	1	0.96	0.98
Thrush	17	80	1	0.88	1	0.94
Lead	71	27	0.74	1	0.64	0.85
Inhibition	98	100	1	1	1	1

Table 8 - MetaData (MD) method results: Training on medium quality/medium quantity corpus, testing on high quality/low quantity corpus.

Term	neg train	pos train	neg test	pos test	P	R	S	F
Development	271	56	98	111	0.67	0.99	0.52	0.80
Spindle	70	48	50	48	0.63	1	0.42	0.77
Nucleus	25	61	99	200	0.85	0.98	0.83	0.91
Transport	102	56	102	91	0.84	1	0.80	0.91
Thrush	42	5	17	80	1	0.88	1	0.94
Lead	202	22	71	27	1	0.22	1	0.36
Inhibition	454	79	98	100	0.96	0.94	0.96	0.95

Table 1: High quality / low quantity corpus: Precision/ Recall/ Specificity / F–measure for the Closest Sense (CS) method on the GO and MeSH test datasets.

**Term**	**Classic distance (only subsumption)**								**Optimized signature + subsumption distance**							
	**Threshold 0.8**				**Threshold 0.7**				**Threshold 0.8**				**Threshold 0.7**			
	P	R	S	F	P	R	S	F	P	R	S	F	P	R	S	F
Development	0.40	0.94	0.59	0.56	0.98	0.78	0.97	0.87	0.98	0.55	0.83	0.71	0.77	0.98	0.79	0.86
Spindle	0.65	1	0.75	0.78	0.90	0.57	0.78	0.70	0.98	1	0.98	0.99	0.96	0.67	0.93	0.79
Nucleus	0.63	0.96	0.56	0.76	0.97	0.82	0.90	0.89	0.94	1	0.88	0.97	0.98	0.91	0.94	0.94
Transport	0.24	0.88	0.46	0.38	0.87	0.80	0.78	0.83	0.32	0.97	0.50	0.48	0.81	0.64	0.54	0.71
Thrush	0.62	1	0.38	0.76	0.89	0.88	0.50	0.88	0.97	0.99	0.89	0.98	1	0.89	1	0.94
Lead	0.30	0.57	0.77	0.39	0.41	0.32	0.75	0.36	0.30	0.57	0.77	0.39	0.74	0.42	0.86	0.53
Inhibition	0.52	0.70	0.62	0.60	0.89	0.52	0.60	0.66	0.85	0.96	0.86	0.90	1	0.73	1	0.84

Table 2: High quality / low quantity corpus: Precision / Recall / Specificity / F–measure for the Term Cooc (TC) method on the GO and MeSH test datasets.

**Term**	**Co-occurrences**				**Hierarchical Cooc**				**Cooc + SVMs**				**Hierarchical Cooc + SVMs**			
	P	R	S	F	P	R	S	F	P	R	S	F	P	R	S	F
Development	0.74	0.74	0.31	0.74	0.70	0.72	0.20	0.71	0.61	0.54	0.91	0.57	0.79	0.78	0.85	0.79
Spindle	0.91	0.90	0.79	0.90	0.83	0.78	0.49	0.80	0.95	0.94	1	0.95	0.98	0.98	1	0.98
Nucleus	0.81	0.80	0.10	0.81	0.80	0.75	0.07	0.78	0.77	0.73	0.90	0.75	0.95	0.95	0.90	0.95
Transport	0.90	0.90	0.60	0.90	0.89	0.89	0.30	0.89	0.90	0.87	0.99	0.88	0.94	0.93	1	0.94
Thrush	0.92	0.83	0.94	0.87	0.82	0.82	0.72	0.82	0.89	0.70	1	0.78	0.89	0.74	1	0.81
Lead	0.89	0.89	0.94	0.89	0.81	0.35	0.50	0.49	0.93	0.92	0.93	0.93	0.80	0.81	0.95	0.81
Inhibition	0.78	0.76	0.62	0.77	0.72	0.55	0.35	0.62	0.85	0.85	0.65	0.85	0.59	0.56	0.85	0.58

Table 3: Medium quality / medium quantity corpus: Precision / Recall / Specificity / F–measure for the Term Cooc (TC) method on the GO and MeSH test datasets.

**Term**	**Co-occurrences**				**Hierarchical Cooc**				**Cooc + SVMs**				**Hierarchical Cooc + SVMs**			
	P	R	S	F	P	R	S	F	P	R	S	F	P	R	S	F
Development	0.76	0.78	0.74	0.77	0.76	0.64	0.66	0.69	0.76	0.77	0.85	0.77	0.78	0.80	0.85	0.79
Spindle	0.76	0.75	0.70	0.75	0.80	0.75	0.62	0.77	0.81	0.77	0.92	0.79	0.81	0.79	0.96	0.80
Nucleus	0.86	0.85	0.51	0.85	0.84	0.84	0.48	0.84	0.80	0.36	0.82	0.50	0.83	0.60	0.93	0.70
Transport	0.89	0.89	0.90	0.89	0.87	0.87	0.87	0.87	0.80	0.75	0.98	0.78	0.83	0.78	0.98	0.80
Thrush	0.73	0.71	0.82	0.72	0.77	0.65	0.71	0.71	0.88	0.15	0.11	0.26	0.75	0.83	0.87	0.78
Lead	0.83	0.80	0.87	0.82	0.84	0.76	0.81	0.80	0.82	0.82	0.80	0.82	0.82	0.82	0.80	0.82
Inhibition	0.74	0.80	0.80	0.77	0.74	0.75	0.74	0.75	0.70	0.61	0.61	0.65	0.69	0.54	0.58	0.61

Table 4: Low quality / high quantity corpus: Precision / Recall / Specificity / F–measure for the Term Cooc (TC) method on the GO and MeSH test datasets.

**Term**	**Co-occurrences**				**Hierarchical Cooc**				**Cooc + SVMs**				**Hierarchical Cooc + SVMs**			
	P	R	S	F	P	R	S	F	P	R	S	F	P	R	S	F
Development	0.68	0.66	0.55	0.67	0.72	0.60	0.46	0.65	0.70	0.71	0.73	0.70	0.74	0.75	0.83	0.74
Spindle	0.79	0.80	0.63	0.79	0.79	0.78	0.45	0.78	0.83	0.76	0.87	0.80	0.88	0.87	0.87	0.87
Nucleus	0.94	0.81	0.77	0.87	0.96	0.95	0.74	0.96	0.94	0.80	0.99	0.86	0.95	0.90	0.99	0.93
Transport	0.69	0.68	0.61	0.69	0.71	0.72	0.52	0.72	0.71	0.63	0.78	0.67	0.72	0.65	0.86	0.68
Thrush	0.54	0.49	0.89	0.52	0.57	0.55	0.74	0.56	0.91	0.91	0.91	0.91	0.43	0.45	0.80	0.44
Lead	0.55	0.53	0.86	0.54	0.53	0.53	0.80	0.53	0.55	0.53	0.78	0.54	0.49	0.51	0.70	0.50
Inhibition	0.75	0.50	0.85	0.60	0.64	0.51	0.75	0.57	0.34	0.34	0.51	0.34	0.26	0.28	0.37	0.27

Table 5: High quality / low quantity dataset with MeSH Text-mined annotations only: Precision / Recall / F–measure for the Term Cooc (TC) method on the GO and MeSH test datasets.

**Term**	**Co-occurrences**			**Hierarchical Cooc**			**Cooc + SVMs**			**Hierarchical Cooc + SVMs**		
	P	R	F	P	R	F	P	R	F	P	R	F
Thrush	0.54	0.25	0.34	0.69	0.46	0.55	0.86	0.83	0.85	0.86	0.83	0.85
Lead	0.72	0.75	0.73	0.69	0.73	0.72	0.80	0.35	0.48	0.81	0.35	0.48
Inhibition	0.24	0.47	0.32	0.37	0.48	0.42	0.80	0.67	0.73	0.80	0.67	0.73

Table 9 - MetaData (MD) method results: Training on low quality/high quantity corpus, testing on high quality/low quantity corpus.

Term	neg train	pos train	neg test	pos test	P	R	S	F
Development	2296	715	98	111	0.67	1	0.50	0.80
Spindle	519	599	50	48	0.65	1	0.46	0.79
Nucleus	131	1336	99	200	0.63	1	0.40	0.77
Transport	1043	699	102	91	0.80	1	0.74	0.88
Thrush	35	1131	17	80	1	0.41	1	0.58
Lead	1564	735	71	27	1	0.07	1	0.14
Inhibition	5247	553	98	100	0.99	0.96	0.99	0.97

Table 10 - Results (precision) of the Close Sense (CS) method tested on the WSD Test Collection (Weeber et al., 2001), with the use of classic distance (only subsumption) and with the use of the optimized signature together with the subsumption distance.

Term	Only subsumption	Optimized
adjustment	0.66	0.66
ganglion	0.92	0.93
extraction	0.62	0.65
japanese	0.65	0.92
pressure	0.98	0.86
surgery	0.61	0.6
depression	0.86	0.96
lead	0.64	0.6
radiation	0.94	0.92
sensitivity	0.98	0.74
transient	0.7	0.97
fat	0.64	0.73
growth	0.85	0.85
man	0.87	0.87
sex	1	0.94
cold	0.8	0.95
fit	0.97	0.69
immunosuppression	0.68	0.84
repair	0.65	0.74
condition	0.79	0.8
implantation	0.97	0.99
strains	0.92	0.94
Average precision	0.8	0.83
